# Prediction models for postoperative recurrence in papillary thyroid carcinoma: a systematic review and critical appraisal

**DOI:** 10.3389/fendo.2026.1831081

**Published:** 2026-07-15

**Authors:** Yunqi Yang, Pengcheng Wang, Lin Zhou, Ruigang Diao, Chenyu Guo

**Affiliations:** Department of Pharmacy, The Affiliated Yantai Yuhuangding Hospital of Qingdao University, Shandong, China

**Keywords:** meta-analysis, papillary thyroid carcinoma, prediction model, recurrence, systematic review

## Abstract

**Background:**

Prediction models for postoperative recurrence in papillary thyroid carcinoma (PTC) have increased substantially in recent years. However, recurrence outcomes are inconsistently defined across studies, particularly with respect to structural and biochemical recurrence, and the quality and clinical applicability of existing models remain uncertain.

**Objective:**

To systematically review and critically appraise multivariable prediction models for structural postoperative recurrence in pathologically confirmed PTC and to evaluate their predictive performance, methodological quality, and risk of bias.

**Methods:**

PubMed, Embase, and the Cochrane Library were searched from inception to February 2026. Studies developing or validating multivariable prediction models for structural recurrence in adult patients with PTC were included. Data extraction was guided by the CHARMS checklist, and risk of bias was assessed using PROBAST. Findings were synthesized narratively, and an exploratory meta-analysis of discrimination performance from validation studies was conducted where appropriate.

**Results:**

Thirteen retrospective studies met the inclusion criteria, all of which were conducted in East Asian populations. Reported discrimination was generally acceptable, with most AUC or C-index values exceeding 0.70. However, all studies were judged to have a high overall risk of bias, primarily due to limitations in the analysis domain, including inadequate handling of overfitting, insufficient sample size justification, limited reporting of missing data, and reliance on internal validation. Only two studies performed external validation. Exploratory pooling of validation AUCs suggested moderate predictive performance but substantial heterogeneity across studies.

**Conclusion:**

Current prediction models for structural recurrence in PTC show promise for individualized risk estimation but remain limited by methodological weaknesses, heterogeneous modelling approaches, inadequate assessment of calibration, and scarce external validation. Future studies should adopt standardized recurrence definitions, improve reporting transparency, and prioritize robust external validation before routine clinical implementation can be recommended.

## 1 Introduction

Thyroid cancer is the most common endocrine malignancy, with global incidence rising rapidly over recent decades (1). This increase is partly attributed to overdiagnosis due to improved imaging, but it also reflects a true increase in risk (2).

Histologically, thyroid cancer is broadly classified into differentiated thyroid carcinoma (DTC), medullary thyroid carcinoma, and anaplastic thyroid carcinoma. DTC includes papillary thyroid carcinoma (PTC), follicular thyroid carcinoma (FTC), oncocytic carcinoma, and the encapsulated follicular variant of PTC (3–5). PTC accounts for >84% of cases and is the primary focus of research and treatment (6).

Most patients with PTC have an excellent prognosis after standard treatment (thyroidectomy, radioactive iodine therapy, and thyroid-stimulating hormone suppression) (7); however, postoperative recurrence and metastasis remain key challenges to long-term survival and quality of life (2). The 10-year recurrence risk in PTC ranges from 7-23% (8, 9). Recurrence often necessitates additional surgery or therapy, thereby increasing healthcare burden, complications, and psychological stress (10, 11). Early identification of high-risk patients is therefore essential for personalized follow-up and management.

Guidelines from the American Thyroid Association (ATA) and other organizations provide risk stratification based on clinicopathological factors (7, 12). However, these systems have limited ability to capture patient heterogeneity and require improved predictive accuracy (13–15). Recent advances in molecular oncology and artificial intelligence have led to the development of predictive models that integrate molecular markers, gene expression profiles, and radiomics features, often using machine learning algorithms (e.g., random forest, support vector machines) to achieve improved performance (16–19). Machine learning-based models in this field are increasingly emphasized to enable precise risk stratification and optimized resource allocation (16, 20–23).

Although several reviews have summarized prediction models for thyroid cancer recurrence, important challenges remain (24). First, recurrence definitions vary substantially across studies, with some investigations including biochemical recurrence or surrogate endpoints that may be influenced by advances in surveillance strategies and diagnostic technologies (25–27). To improve outcome consistency and clinical relevance, the present review focused specifically on structural postoperative recurrence in papillary thyroid carcinoma. Second, the number of prediction model studies has increased rapidly in recent years, particularly with the emergence of machine learning-based approaches and radiomics-driven models, generating a growing body of evidence that has not yet been comprehensively synthesized. Finally, substantial heterogeneity persists in study design, predictor selection, modelling strategies, and validation procedures, making it difficult to assess the overall performance and clinical applicability of existing models.

Therefore, we conducted an updated systematic review of prediction models for structural recurrence (STR) in papillary thyroid carcinoma. In addition to summarizing model characteristics and methodological quality, we performed an exploratory meta-analysis of discrimination performance from validation studies to provide a quantitative assessment of current predictive performance and to identify priorities for future model development and validation.

## 2 Methods and design

This systematic review was conducted and reported in accordance with the Preferred Reporting Items for Systematic Reviews and Meta-Analyses (PRISMA) statement (28). The protocol was prospectively registered in International Prospective Register of Systematic Reviews (PROSPERO) (registration number: CRD420251229374).

### 2.1 Search strategy and study selection

A systematic search was conducted in PubMed, Embase, and the Cochrane Library from database inception to February 2026. Search strategies combined controlled vocabulary (MeSH in PubMed; Emtree in Embase) and free-text terms related to thyroid carcinoma, recurrence, and prediction modelling (e.g., “thyroid neoplasm”, “recurrence”, “predictive model”). No restrictions were applied with respect to country, region, language, publication year, or clinical setting. Detailed search strategies are provided in the supplementary materials.

### 2.2 Eligibility criteria

Studies were included if they met the following criteria based on the PICOS framework:

1. Population: Adult patients (≥18 years) with pathologically confirmed PTC who underwent curative-intent surgery (total thyroidectomy or lobectomy).
2. Intervention(s) or exposure(s): Multivariable clinical prediction models (including but not limited to nomograms, scores, or algorithms) incorporating at least two predictors to estimate individual risk of PTC recurrence.
3. Comparator(s): Not required; studies with or without model comparisons were eligible.
4. Outcome(s): Primary: postoperative recurrence (local, regional, or distant), confirmed by pathology or imaging (excluding structural incomplete response or biochemical incomplete response).
  Secondary: recurrence-free survival (RFS).
5. Timing and Setting: No restrictions on prediction horizon or clinical setting.

### 2.3 Exclusion criteria

Studies were excluded if they:

2.3.1 Included pediatric patients, mixed age groups, or non-PTC thyroid cancers (follicular, medullary, or anaplastic)

2.3.2 Were non-original research (e.g., reviews, meta-analyses, guidelines, editorials) or database-based studies

2.3.3 Focused solely on single risk factors rather than multivariable models

2.3.4 Provided insufficient data (e.g., lacking baseline characteristics or model details)

### 2.4 Study selection

**Figure 1:**
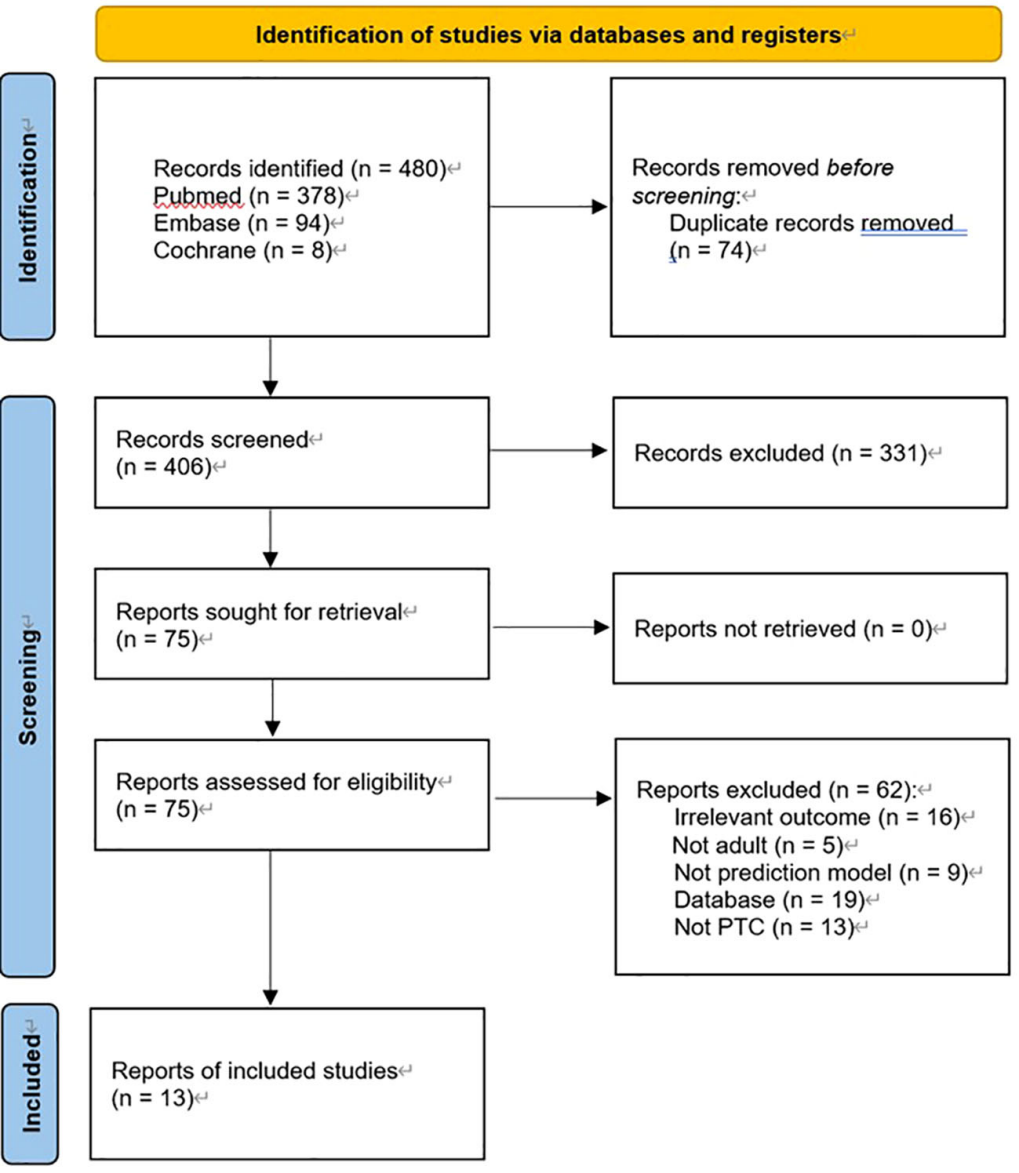
Preferred reporting Items for systematic reviews and meta-analyses (PRISMA) flowchart of literature searching and selection.

Records were deduplicated using reference management software. Two reviewers independently screened titles and abstracts, followed by full-text assessment according to predefined eligibility criteria. Disagreements were resolved through discussion or consultation with a third reviewer. The study selection process followed Preferred Reporting Items for Systematic Reviews and Meta-Analyses (PRISMA) guidelines and is illustrated in **Figure 1**.

### 2.5 Data extraction

Two reviewers independently extracted data using a standardized form based on the CHARMS checklist (29). Extracted items included:

Study characteristics (first author, year, country, design);

Participant details (sample size, age, follow-up duration, recurrence definition);

Model details (type, predictors, method).

Performance metrics (discrimination: C-index/AUC with 95% CI; calibration: plots, slope; clinical utility: decision curve analysis).

When multiple models were reported, only the best-performing model (as designated by the authors) was extracted for synthesis.

### 2.6 Quality assessment of literature

Risk of bias and applicability were assessed using PROBAST, which evaluates four domains of bias (participants, predictors, outcome, and analysis) and three domains of applicability. Each domain was rated as low risk, high risk, or unclear risk according to PROBAST signaling questions. An overall high risk of bias was assigned when at least one domain was rated as high risk. Assessments were performed independently by two reviewers, with discrepancies resolved through discussion or consultation with a third reviewer.

### 2.7 Synthesis of results

When multiple models were reported within a study, only the primary or final model identified by the authors was extracted to avoid duplicate inclusion.

Given the anticipated substantial clinical and methodological heterogeneity across studies—including differences in outcome definitions, prediction horizons, modelling techniques, predictor sets, and validation strategies—narrative synthesis was adopted as the primary analytical approach.

Discrimination performance from validation analyses was summarized descriptively in the main figures using AUC values on the original scale with corresponding 95% confidence intervals. To complement the narrative synthesis, a secondary exploratory meta-analysis was conducted when a sufficient number of studies reported AUC estimates from validation analyses. For pooling purposes only, AUC values were transformed to the logit scale to improve statistical properties, and random-effects models were applied. Between-study heterogeneity was quantified using the I² statistic.

As most included studies relied on internal validation and showed variability in outcome definitions and prediction timeframes, meta-analytic results were interpreted strictly as exploratory and descriptive. These findings were used solely to supplement the narrative synthesis and were not intended to support conclusions regarding model generalizability or clinical applicability.

## 3 Result

### 3.1 Selection of studies

In the final search, 480 records were identified and organized in tabular format. These records were independently screened by two investigators to exclude studies that clearly did not meet the inclusion criteria. Ultimately, 13 articles were included based on the predefined eligibility criteria. The study selection process was presented using the PRISMA flow diagram.

### 3.2 Study designs and study populations

The basic study characteristics are shown in **Table 1**. Among the 13 included studies, 11 were conducted in China, 1 in South Korea, and 1 in both South Korea and China. All studies were retrospective in design. The sample sizes used for model development and validation ranged from 194 to 3,575.

**Table 1:** Characteristics of the studies included in the systematic review.

Author, year	Country	Study design	Enrolment period	Sample size (development/validation)	Age of participants (median)/mean ± SD/%
Hu et al. (2024) (30)	China	Retrospective cohort	September 2017 to May 2020	811	<55 87.92% ≥55 12.08%
Lang et al. (2015) (31)	China and South Korea	Retrospective cohort	Development set: 1970-2006 Validation set: 1998-2004	1124 (849/275)	45.6 ± 16.8 (D) 49.2 ± 13.2 (V)
Lee et al. (2024) (32)	South Korea	Retrospective cohort	January 2006 to December 2021	1613	48.24 ± 0.57
Li et al. (2023) (33)	China	Retrospective cohort	September 2017 to May 2020	955 (671/284)	39.878 ± 11.568
Lu et al. (2022) (34)	China	Retrospective cohort	January 2010 to December 2014	361 (253/108)	21-79 (42)
Ma et al. (2023) (35)	China	Retrospective cohort	December 2012 to December 2017	719 (617/102)	42.94 ± 14.35(D)* 38.24 ± 14.21(V)
Pang et al. (2025) (36)	China	Retrospective cohort	November 2017 to March 2023	194	41.73 ± 11.69
Song et al. (2025) (37)	China	Retrospective cohort	January 2010 to December 2022	418 (293/125)	<55 66.5% ≥55 33.5%
Wang et al. (2024) (38)	China	Retrospective cohort	January 2009 to December 2018	2242 (1795/449)	- (42)
Xu et al. (2021) (39)	China	Retrospective cohort	1996-2009	245	18-70 (41)
Xu et al. (2023) (17)	China	Retrospective cohort	January 2012 to June 2015	280 (169/111)	40.74 ± 13.61
Yuan et al. (2024) (18)	China	Retrospective cohort	January 2012 to December 2016	3575 (2503/1072)	44.8 ± 11.7
Zhou et al. (2024) (19)	China	Retrospective cohort	July 2017 to August 2021	1124 (849/275)	50.7 ± 11.7

### 3.3 Definition of recurrence outcome

This review exclusively included studies using recurrence prediction as the outcome. Reported outcomes included recurrence, structural recurrence, disease-free survival, and lymph node recurrence. The timing of recurrence assessment varied across studies; however, all reported a follow-up duration exceeding 12 months. All studies focused on recurrence in papillary thyroid carcinoma, and two articles specifically analyzed different histological subtypes. In addition, information regarding initial treatment was extracted, including lobectomy or total thyroidectomy and whether lymph node dissection was performed. Considerable heterogeneity was observed across studies in outcome definitions, including disease-free survival (DFS) at 1, 3, and 5 years, RFS at 3 years, neck lymph node recurrence, lateral neck lymph node recurrence, and structural recurrence.

### 3.4 Predictor

**Figure 2:**
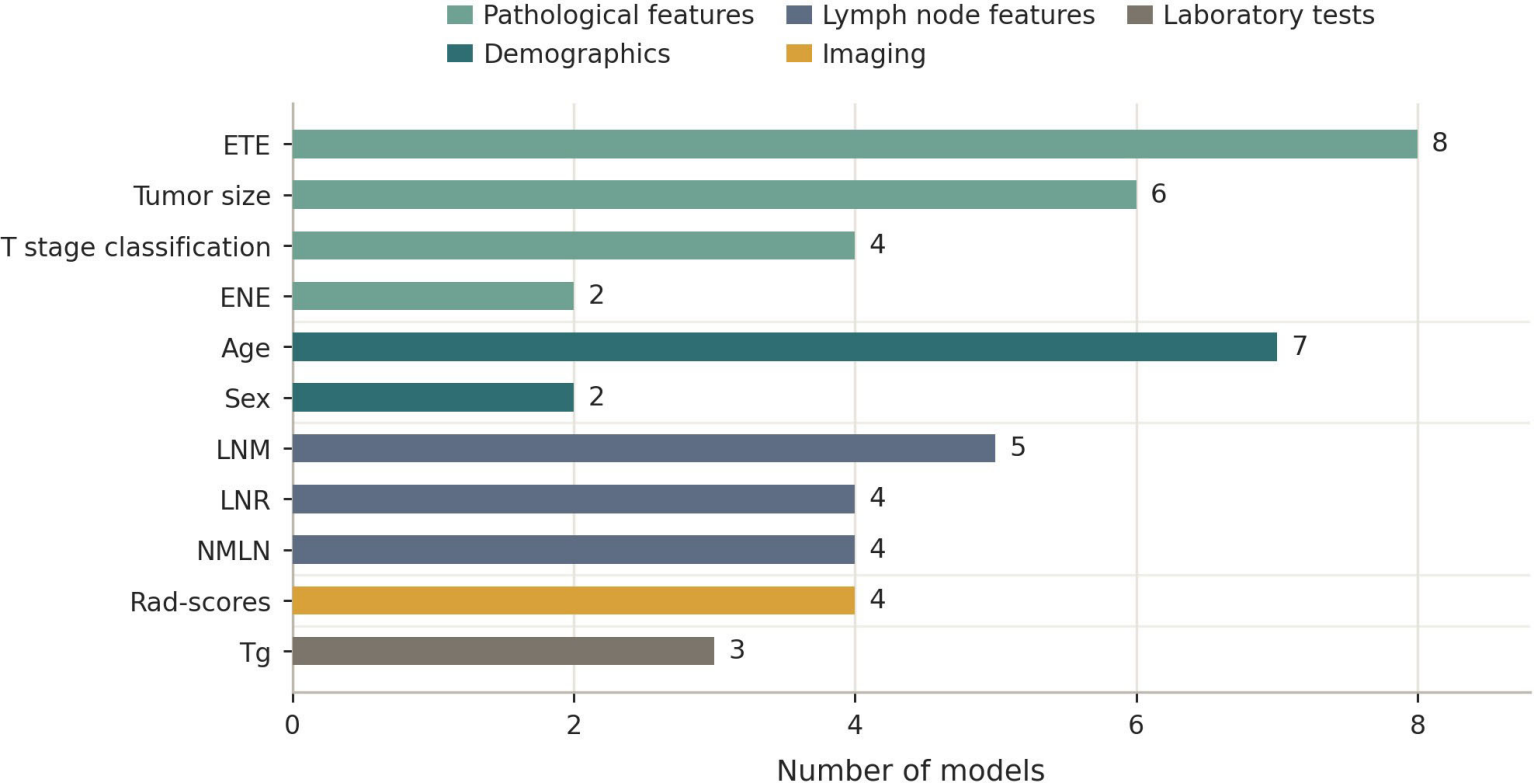
The predictive factors that appear more than twice in the PTC prediction model.

Predictors were categorized into five groups: demographic characteristics, pathological characteristics, lymph node characteristics, imaging findings, and laboratory test results. All variables incorporated into the predictive models are listed in **Table 2**. **Figure 2** presents variables reported in more than two studies, including age, sex, tumor size, tumor stage or grade, extranodal extension (ENE), lymph node metastasis (LNM), number of lymph node metastases, lymph node metastasis ratio (LNR), number of metastatic lymph nodes (NMLN), radiomics scores (Rad-scores), and thyroglobulin (Tg). B-Raf proto-oncogene (BRAF) mutation, a molecular marker with established prognostic value, was included as a predictor in one study. Another study stratified patients according to BRAF status and developed separate predictive models for analysis. Most models were developed using clinical predictors alone, while only a minority incorporated radiomics features in combination with clinical variables. No imaging-only prediction models were identified. **Table 2** shows that all models included clinical predictors, and four studies additionally integrated imaging-derived radiomics features with clinical variables.

**Table 2:** Prediction models information for PTC recurrence.

Author (year)	Patient	Predictor modality	Treatment	Outcome	Final predictors number	Predictors	Mean time of follow-up
Hu et al. (2024) (30)	PTC	Clinical	Surgery	1, 3-, and 5-year DFS	6	BRAF mutation-negative: ETE, vascular tumor thrombus, lymph node yield BRAF mutation-positive: ENE, NMLN, vascular tumor thrombus, pathological stage	41.52 months (IQR, 32.97–57.60)
Lang et al. (2015) (31)	PTC	Clinical	Total thyroidectomy	10-year recurrence	6	Age, sex, tumor size, tumor multifocality, ETE, nodal status	17.1 years (range: 7.2-47.2 years) (D) 10.7 years (range: 9.7-26.1 years) (V)
Lee et al. (2024) (32)	PTC	Clinical	Thyroid lobectomy or total thyroidectomy	Recurrence	12	Age, sex, tumor size, tumor multiplicity, ETE, ENE, Tumor classification, node classification, FreeT4, Tg, Thyroid-stimulating hormone, triiodothyronine	>5 years after the surgery
Li et al. (2023) (33)	PTC	Clinical	Surgery	Recurrence	4	Age 55, ENE>0.5, LNR, initial treatment	/
Lu et al. (2022) (34)	PTC	Combined (clinical + imaging)	Surgery	Recurrence	3	Age, LNM, Rad-scores	108 months (range 8–137 months)
Ma et al. (2023) (35)	N1a PTC	Clinical	Total thyroidectomy and central lymph node dissection	Structural recurrence	5	Tumor size, extrathyroidal infiltration, BRAF state, LNM, LNR	69.69 ± 17.07 months (T) 52.19 ± 19.36 months (V)
Pang et al. (2025) (36)	PTC	Combined (clinical + imaging)	Total thyroidectomy	Cervical Lymph Node Recurrence	5	Rad-scores, age, Tg, TgAb, Tumor stage	/
Song, et al. (2025) (37)	T4a PTC	Clinical	Surgery	1-, 3-, and 5-year DFS	4	Age, LNM, vocal cord paralysis, microvascular invasion	50 months (range 4–149 months)
Wang et al. (2024) (38)	PTC	Clinical	Surgery	Structural recurrence	9	Tg, LNR, node stage, comorbidity of hypertension, LDL, BMI, number of LNs dissected, comorbidity of diabetes, LN dissection	45.5 months (range: 12.0-142.7 months)
Xu et al. (2021) (39)	PTC	Clinical	Surgery and central lymph node dissection	Lateral neck lymph node recurrence	3	Tumor size, ETE, NMLN	12–170 Month after surgery
Xu et al. (2023) (17)	PTC	Combined (clinical + imaging)	Surgery	Recurrence	7	Rad-scores, age, NMLN, LN status, tumor stage, presence of bilaterality, multifocality	68 months (range: 6–112 months).
Yuan et al. (2024) (18)	PTC	Clinical	Surgery	3-, 4-year DFS	5	Triglycerides, high-density lipoprotein, tumor size, LNM, ETE	56.7 months (IQR, 43.0-73.0)
Zhou et al. (2024) (19)	PTC	Combined (clinical + imaging)	Total thyroidectomy	3-year RFS	6	Rad-scores and tumor size, ETE, LNM, capsular invasion, Tg	23 months, range 12–62 months)

*T is Training set, V is Validation group; BMI, body mass index; DFS, Disease-free survival; ENE, extranodal extension; ETE, extrathyroidal extension; FreeT4, free thyroxine; IQR, interquartile range; LDL, low-density lipoprotein; LN, lymph node; LNM, lymph node metastases; LNR, lymph node metastasis ratio; N1a, N1a-stage papillary thyroid carcinoma; NMLN, number of metastatic lymph node; Rad-scores, Radiomics scores; RFS, recurrence-free survival; STR, structural recurrence; Tg, thyroglobulin; TgAb, thyroglobulin antibody.

### 3.5 Prediction models for recurrence of papillary thyroid carcinoma

Concerns regarding applicability mainly arose from heterogeneity in outcome definitions. Several studies defined recurrence exclusively as cervical lymph node recurrence, whereas this review focused on overall postoperative structural recurrence. Therefore, although these models may be internally valid, their applicability to the review question is limited.

### 3.6 Model performance and validation characteristics

Thirteen studies developed prediction models using different modelling approaches. Six models used Cox proportional hazards regression, four used logistic regression, and three used machine learning-based approaches, including one multimodal deep learning model.

All studies performed internal validation, while only two additionally conducted external validation. For internal validation, eight models used random data splitting, three used bootstrap resampling, and two applied cross-validation techniques. Except for the three machine learning-based models, the remaining ten models were presented as nomograms.

Most studies assessed model calibration using calibration plots, although two (32, 39) did not report any calibration assessment. Clinical utility was evaluated with decision curve analysis in seven studies, whereas five did not assess clinical utility. Only one study (32) reported applying its prediction model in a real-world clinical setting.

Model discrimination was primarily assessed using the area under the receiver operating characteristic curve (AUC or AUROC). Ten studies reported AUC/AUROC values; among these, nine models achieved an AUC greater than 0.7 in validation analyses, while one model reported an AUC below 0.7. Three studies assessed discrimination using the concordance index (C-index), with all reported values exceeding 0.7.

Due to substantial heterogeneity in outcome definitions, prediction time horizons, modelling approaches, and validation strategies, a quantitative meta-analysis of model discrimination was not performed.

### 3.7 Risk of bias and applicability assessment

**Figure 3:**
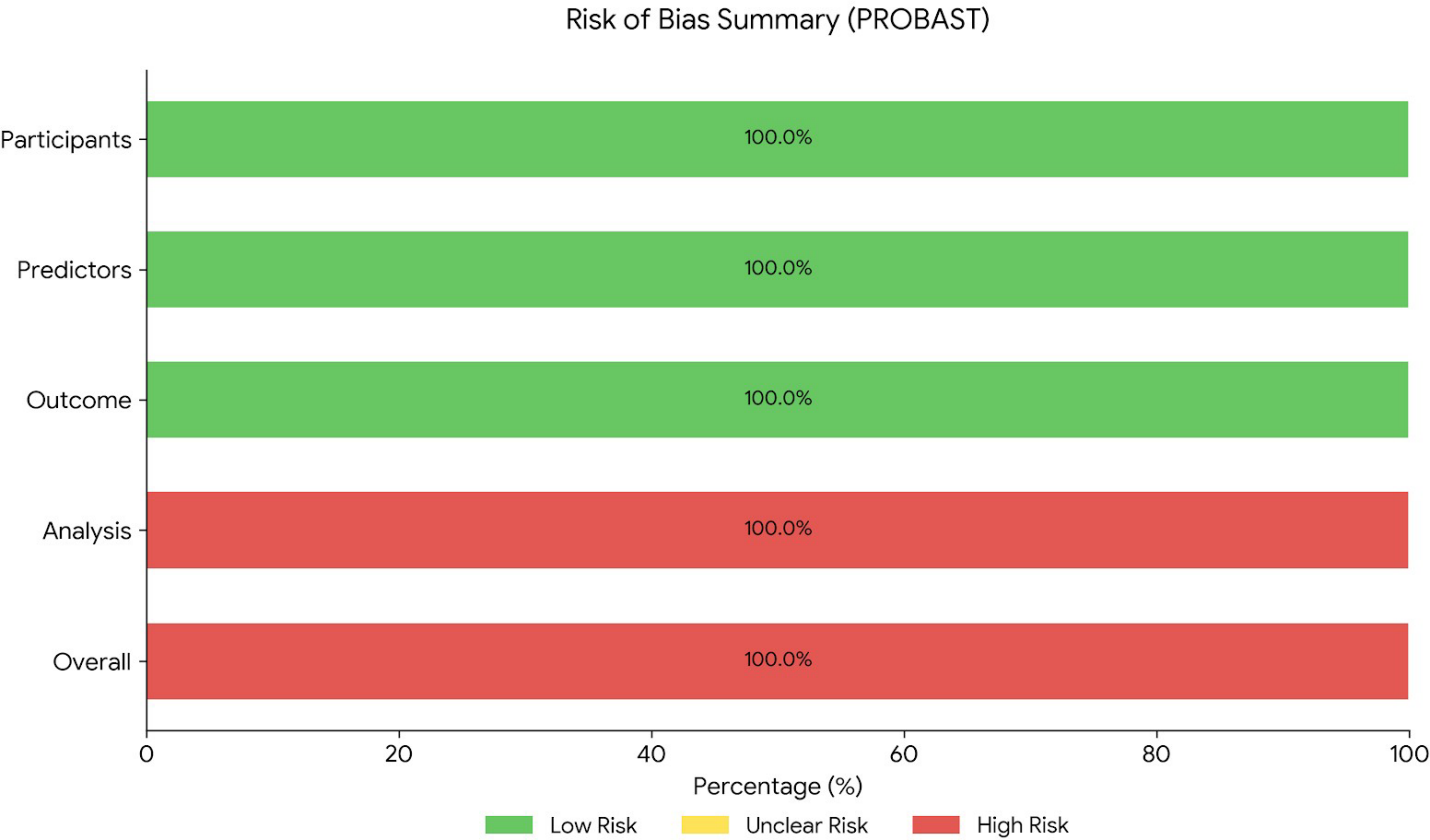
Risk of bias assessment using the PROBAST based on four domains.

Risk of bias was assessed using PROBAST. All included studies were rated as having an overall high risk of bias (**Figure 3**). Risk was low across the participants, predictors, and outcome domains, but high in the analysis domain in all studies, mainly due to inadequate handling of overfitting (e.g., reliance on simple random splitting), limited use of robust internal validation methods (e.g., bootstrapping), and insufficient reporting of sample size justification or missing data.

**Figure 4:**
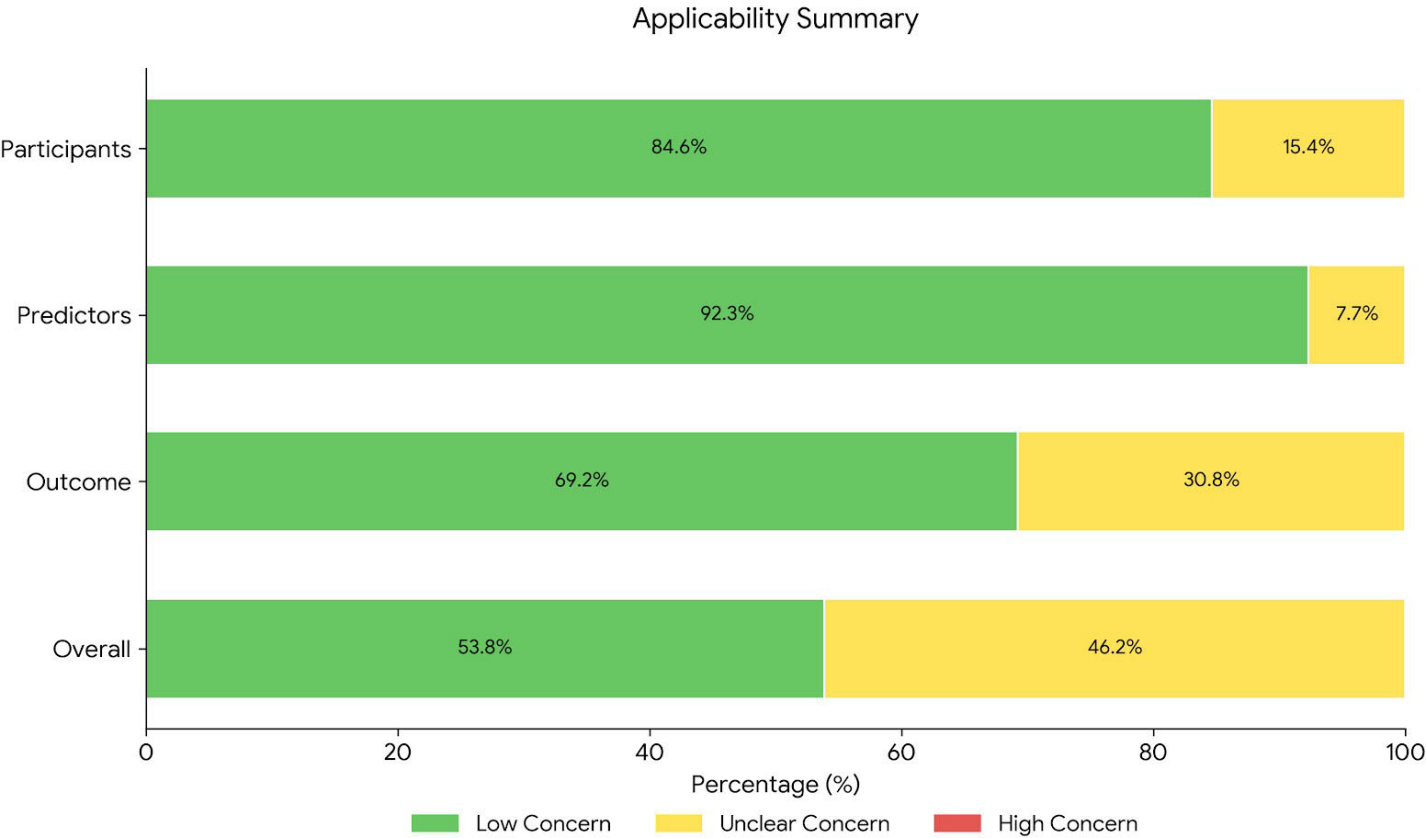
Applicability assessment based on models for all included models (N = 13).

Applicability concerns were generally low across domains (**Figure 4**). Minor concerns in a few studies were related to specialized predictors (e.g., radiomics requiring specific protocols) or narrow outcome definitions. Overall, the models were considered reasonably applicable to predicting structural recurrence in unselected PTC patients.

Applicability specific to the review question was examined (**Table 3**):

**Table 3:** Characteristics of the models for PTC recurrence.

Author (year)	Model presentation	Modelling method	Selection of candidate predictors	Selection of final predictors	Model performance (95%CI)	Internal validation	External validation	Calibration	Clinical utility
Traditional statistical models									
Hu et al. (2024) (30)	Nomogram	Cox regression	All available predictors	LASSO selection	AUC BRAFneg model: 1 year: 0.94; 3 years: 0.89; 5 years: 0.86 C index BRAFneg model: 0.880 (0.788-0.972)	Bootstrap	/	Calibration plot	DCA
Lang et al. (2015) (31)	Nomogram	Multilevel logistic regression	Based on prior knowledge	Stepwise selection	AUC Training cohort: 0.800 (0.763-0.837) Validation group: 0.743 (0.658- 0.828)	Cross-validation	Temporal	Calibration plot	/
Li et al. (2023) (33)	Nomogram	Logistic regression	Based on prior knowledge	LASSO selection	AUC Training cohort: 0.819 (0.729-0.909) Validation group: 0.818 (0.670-0.909)	Random split data	/	Calibration plot	DCA
Lu et al. (2022) (34)	Nomogram	Cox regression	Based on univariable associations	LASSO selection	C-Statistic Training group: 0.829 Validation group: 0.845	Random split data	/	Calibration plot	DCA
Ma et al. (2023) (35)	Nomogram	Logistic regression	All available predictors	LASSO selection	AUC Training group: 3 years: 0.701 5 years: 0.702 Validation group: 3 years: 0.576 5 years: 0.599	Bootstrap	Geographical	Calibration plot	DCA
Xu et al. (2021) (39)	Nomogram	Cox regression	All available predictors	Pre-specified model (not selection)	AUC Training group: 0.790 (0.709-0.871) Validation group: 0.801	Bootstrap	/	/	/
Yuan et al. (2024) (18)	Nomogram	Cox regression	Based on prior knowledge	/	C index Training group: 3 years: 0.80 (0.73-0.87) 4 years: 0.82 (0.73-0.90) Validation group: 3 years: 0.90 (0.84-0.96) 4 years: 0.88 (0.81-0.94)	Random split data	/	Calibration plot	DCA
Song, et al. (2025) (37)	Nomogram	Cox regression	Based on prior knowledge	Backward elimination	C-index Training set: 0.778 (0.700-0.857) Validation set: 0.793 (0.654-0.933)	Random split data	/	Calibration plot	/
Machine learning model									
Lee, et al. (2024) (32)	Multimodal Deep Learning Model	/	Based on prior knowledge	Pre-specified model (not selection)	AUROC 0.9622 (0.9251–0.9812)	Cross-validation	/	/	Real-time Prediction
Pang et al. (2025) (36)	Nomogram	Machine learning techniques	All available predictors	LASSO selection	AUC Combined model: 0.94	Random split data	/	Calibration plot	DCA
Wang et al. (2024) (38)	Multiple machine learning models	Machine learning techniques	Based on univariable associations	LASSO selection	AUC RF: 0.766 (0.702-0.845)	Random split data	/	Calibration curve	/
Xu et al. (2023) (17)	Nomogram	Logistic regression	All available predictors	LASSO selection	AUC Support Vector: 0.754 (0.649-0.859)	Random split data	/	Calibration plot	/
Zhou et al. (2024) (19)	Nomogram	Cox regression	Based on prior knowledge	Pre-specified model (not selection)	AUC Training group: 0.851 (0.788 - 0.913) Validation group: 0.885 (0.805 - 0.930)	Random split data	/	Calibration plot	DCA

Outcome: Unclear in Siyuan Xu (39), Hongxi Wang (38), and Feng Pang (36), due to site-specific recurrence focus or unclear distinction between structural and biochemical recurrence (Feng Pang (36) was more diagnostic than prognostic).

Participants: Unclear in Teng Ma (35) and Yixuan Song (37) due to highly selected subgroups (e.g., clinical N1 disease (cN1) disease or recurrent laryngeal nerve invasion).

Studies by Bin Lu (34), Haijun Xu (17), and Brian Lang (2015) showed high applicability, with cohorts and outcomes closely aligned with the review criteria.

### 3.8 Meta-analysis

**Figure 5:**
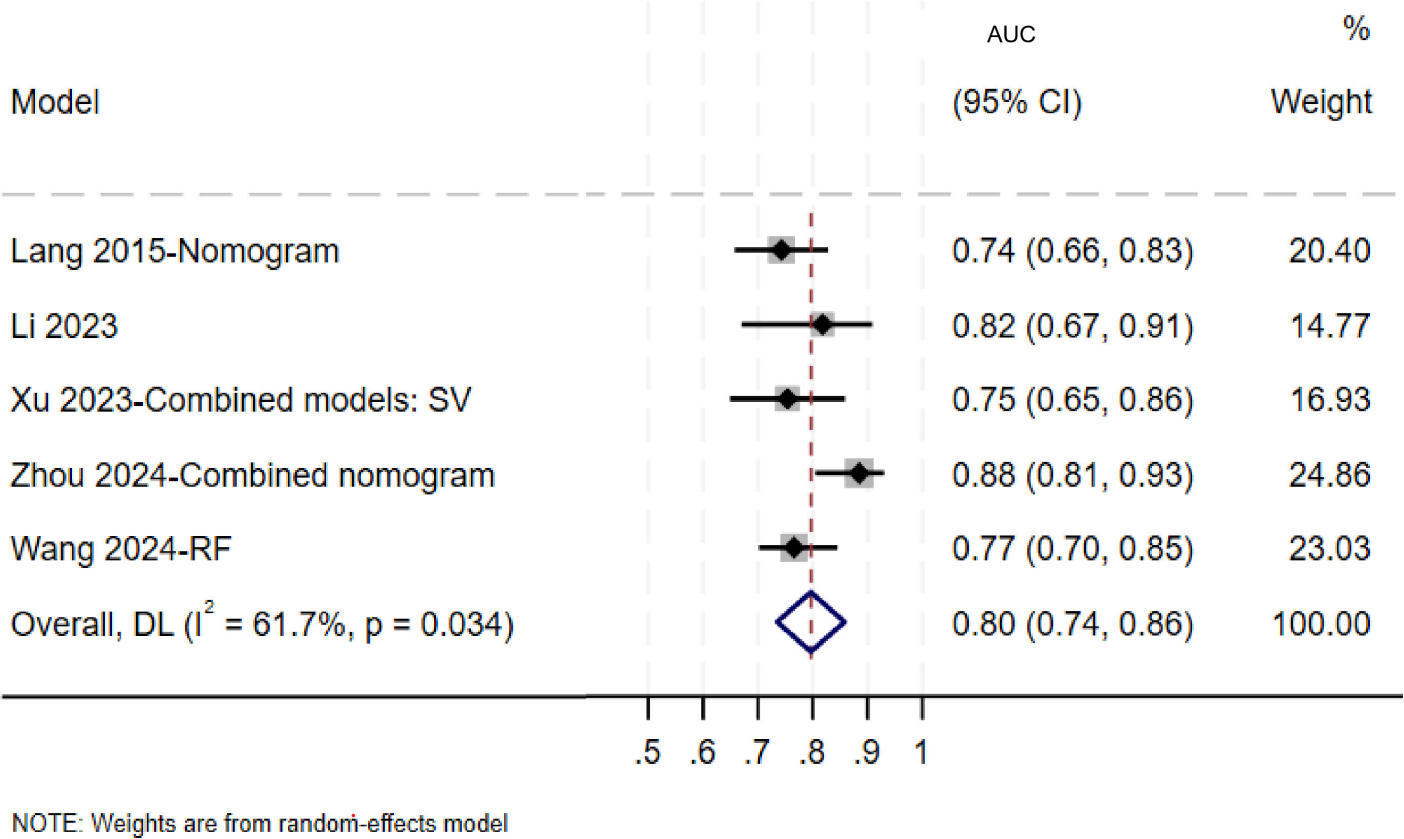
Forest plot of the random effects meta-analysis of pooled AUC estimates for 5 validation models.

Five studies (17, 19, 31, 33, 38) reporting AUC estimates from validation analyses were eligible for the exploratory meta-analysis. These models included conventional nomograms, combined clinical-radiomics models, and machine learning-based approaches. Reported AUC values ranged from 0.743 (95% CI 0.658-0.828) to 0.885 (95% CI 0.805-0.930), indicating moderate to good discriminative performance across studies, as shown in **Figure 5**.

For meta-analytic purposes, AUC values were pooled on the logit scale using a random-effects model. The pooled estimate indicated overall moderate discriminative ability; however, substantial between-study heterogeneity was observed (I² = 61.7%). This heterogeneity likely reflects differences in outcome definitions, prediction horizons, modelling strategies, and validation approaches.

As all included AUC estimates were derived from internal validation analyses, the pooled result is likely to be optimistically biased and should not be interpreted as evidence of model transportability or clinical applicability. Therefore, the meta-analysis was conducted as an exploratory analysis and is presented only to supplement the primary narrative synthesis.

## 4 Discussion

### 4.1 Principal findings

This systematic review identified and critically appraised prediction models developed to estimate postoperative recurrence risk in PTC. Thirteen studies met the eligibility criteria. All included studies were retrospective and conducted in East Asian populations, primarily in China and South Korea. Although no geographical restrictions were applied during the search or study selection process, the eligible evidence base was geographically concentrated. A previous study has also highlighted similar geographical limitations in this research area (24). This finding is important because model performance in one healthcare system may not be directly transferable to another, where surgical practices, pathology reporting standards, imaging protocols, follow-up intensity, and patient characteristics may differ.

Most models demonstrated moderate to good discrimination in internal validation, with reported AUC or C-index values generally exceeding 0.70. Frequently selected predictors were largely consistent with established clinicopathological risk factors, including age, tumor size, ETE, lymph node metastasis, lymph node metastasis ratio, ENE, and Tg. Several models additionally incorporated radiomics-derived features (17, 19, 34, 36), and a small number used machine learning (17, 19, 36) or multimodal deep learning (32, 38) approaches.

However, acceptable discrimination should not be interpreted as evidence of clinical readiness. Using PROBAST (29), all included studies were rated as having a high overall risk of bias, mainly due to weaknesses in the analysis domain. Common issues included inadequate control of overfitting, reliance on simple random data splitting, insufficient sample size justification, incomplete reporting of missing data handling, limited assessment of calibration, and scarce external validation. Only two studies (31, 35) evaluated model performance using independent external datasets. These limitations indicate that current models should be considered promising research outputs rather than tools suitable for routine clinical decision-making.

The exploratory meta-analysis of validation AUC values suggested moderate pooled discrimination, but substantial heterogeneity was observed. This heterogeneity likely reflects differences in recurrence definitions, prediction horizons, modelling strategies, predictor sets, validation methods, and clinical settings. As the pooled estimates were derived mainly from internally validated models, they should be interpreted as descriptive evidence only. They do not support conclusions regarding model transportability or clinical applicability in routine practice.

### 4.2 Contributions of this review

This review contributes to the literature in several ways. First, it focuses specifically on multivariable prediction models for postoperative recurrence in pathologically confirmed PTC. This distinguishes it from broader reviews addressing thyroid cancer prognosis, single risk factors, diagnostic models, or treatment response prediction (24, 40, 41). This distinction is clinically relevant because prediction of postoperative recurrence is directly associated with long-term surveillance strategies, adjuvant treatment decisions, imaging follow-up, and patient counselling.

Second, this review emphasizes the importance of outcome definition. Across included studies, recurrence was variably defined as structural recurrence, cervical lymph node recurrence, lateral neck LN recurrence, DFS, RFS, or time-specific recurrence. These outcomes are related but not interchangeable. A model predicting five-year disease-free survival cannot be interpreted in the same way as a model predicting confirmed structural recurrence or lateral neck lymph node recurrence. Differences in event definitions, time origin, follow-up duration, and prediction horizons may substantially influence both recurrence estimates and apparent model performance.

Third, this review provides a structured methodological appraisal using CHARMS and PROBAST. Although many models reported favorable discrimination, all studies were judged to have a high overall risk of bias. This discrepancy is clinically important. A high AUC may suggest good performance, but it does not ensure accurate absolute risk estimation, robustness in external populations, or clinical benefit. Calibration, overfitting control, missing data handling, complete model reporting, clinical utility assessment, and external validation are equally essential.

Fourth, this review highlights the persistent gap between model development and clinical implementation. Although numerous prediction models have been proposed, only a limited number have progressed beyond initial development and internal validation. From a clinical perspective, the critical issue is not whether a model performs well in the dataset from which it was derived, but whether it can maintain reliable performance and support decision-making across independent institutions, diverse patient populations, and real-world clinical settings. At present, evidence regarding such external applicability and clinical utility remains limited.

### 4.3 Interpretation of predictors and model structures

The predictors included in the identified models largely correspond to factors that are well established in clinical practice for managing PTC. Age, tumor size, extrathyroidal extension, lymph node metastasis, lymph node metastasis ratio, extranodal extension, and Tg are biologically plausible and clinically relevant predictors of recurrence. Their repeated selection across different models supports their importance in postoperative risk estimation.

The prominent role of lymph node-related variables is particularly notable. PTC recurrence frequently involves cervical lymph nodes, and the extent of nodal disease may provide more prognostic information than a simple binary classification of node-positive versus node-negative status. Variables such as lymph node metastasis ratio, NMLN, ETE, and nodal compartment involvement may better characterize both disease burden and biological aggressiveness. Future models should therefore avoid oversimplifying nodal status into binary variables when more detailed nodal information is available.

Compared with conventional staging or risk stratification systems, multivariable prediction models may provide more individualized estimates of recurrence risk. This may be particularly relevant for patients in intermediate-risk categories, in whom decisions regarding surveillance intensity, radioactive iodine therapy, and imaging follow-up are often complex. In clinical practice, these are the patients for whom more precise risk estimation is most needed. However, whether current models improve decision-making beyond established clinicopathological systems remains uncertain, as direct comparative studies and prospective impact evaluations are still lacking.

Radiomics features were incorporated into several models, typically in combination with clinical variables. These models sometimes reported higher discrimination, suggesting that quantitative imaging features may capture tumor heterogeneity not fully reflected by conventional clinicopathological variables. However, radiomics-based models also introduce important practical challenges. Their performance depends on imaging acquisition protocols, segmentation methods, feature extraction pipelines, and analytical decisions. Without standardization and external validation of reproducibility, strong performance in development cohorts may not translate into consistent performance across institutions.

Molecular predictors were infrequently included. Although BRAF V600E (42–44) and other molecular alterations have been widely studied in thyroid cancer, their incremental value in predicting postoperative recurrence remains uncertain. Molecular markers should not be incorporated into prediction models solely based on biological plausibility. Their utility should be demonstrated through improved discrimination, better calibration, meaningful reclassification, and enhanced clinical utility beyond established clinicopathological predictors.

Machine learning-based models were relatively uncommon among the included studies. This does not imply that machine learning is unimportant in thyroid cancer research. Many machine learning studies (16, 45–48) focus on diagnosis, ultrasound classification, cytological interpretation, malignancy risk stratification, or treatment response, and therefore fall outside the scope of this review. For models specifically designed to predict postoperative recurrence in confirmed PTC, machine learning may help capture nonlinear relationships and complex interactions. However, these potential advantages are accompanied by risks, including overfitting, poor calibration, limited interpretability, and reduced transportability, particularly in small single-center retrospective datasets. Current evidence does not demonstrate that machine learning models consistently outperform well-developed conventional regression models for this clinical task.

### 4.4 Relationship with preoperative thyroid cytology risk stratification

The Bethesda System for Reporting Thyroid Cytopathology (TBSRTC) provides a standardized framework for the preoperative evaluation and risk stratification of thyroid nodules and has contributed substantially to individualized clinical decision-making (49). The recently released third edition (2023) represents a notable refinement, particularly by standardizing the terminology for Category III as “Atypia of Undetermined Significance (AUS)” and recommending subclassification into nuclear (AUS-N) and other (AUS-O) atypia (50, 51). This refinement recognizes the biological heterogeneity within Category III, in which AUS-N is associated with a significantly higher risk of malignancy (ROM) and potentially more aggressive behavior compared with AUS-O.

While TBSRTC primarily stratifies preoperative malignancy risk, its implications extend to postoperative recurrence prediction. However, this review highlights a critical limitation: most existing prediction models treat Bethesda categories as homogeneous entities and do not incorporate these finer cytological subtypes. This omission may partly contribute to heterogeneity in model performance, as the true risk associated with a nodule is not fully captured by broad categorical labels.

Furthermore, preoperative cytology and postoperative tumor staging represent distinct but complementary dimensions of risk assessment. Although tumor stage remains the gold standard for prognosis, its distribution is often insufficiently reported in model development studies. Future research should adopt a “continuum of care” framework by integrating detailed preoperative cytological information (e.g., AUS subtypes, molecular markers) with postoperative pathological staging. Such an integrated approach would likely improve both the transportability and precision of recurrence prediction models, thereby bridging the gap between diagnosis and long-term surveillance.

### 4.5 Methodological limitations of existing models

Several methodological limitations were consistently identified across the included studies.

First, external validation was insufficient. Although all studies performed some form of internal validation, most relied on random data splitting, bootstrap resampling, or cross-validation within the same source population. These approaches are useful during model development but do not establish generalizability across different institutions, populations, imaging protocols, surgical strategies, pathology practices, and follow-up systems. External validation remains the most critical step before a model can be considered for clinical application.

Second, recurrence outcomes were heterogeneous. Some studies predicted structural recurrence, whereas others assessed cervical lymph node recurrence, lateral neck lymph node recurrence, DFS, RFS, or time-specific recurrence. These definitions may reflect different clinical questions and should not be directly pooled, compared, or treated as equivalent. Future studies should clearly define postoperative recurrence, distinguish recurrence from persistent disease and biochemical incomplete response, specify the method of event confirmation, and clearly report the prediction horizon.

Third, overfitting was often insufficiently addressed. Several studies used relatively small or moderate-sized retrospective datasets while evaluating multiple candidate predictors. Sample size justification and events-per-predictor considerations were frequently incomplete. In prediction modeling, apparent performance may be overly optimistic when model complexity exceeds the available information in the data. Penalization methods, shrinkage techniques, bootstrap optimism correction, and external validation should be applied more consistently.

Fourth, calibration was incompletely reported. Discrimination metrics such as AUC and C-index describe how well a model separates patients who experience recurrence from those who do not. However, they do not reflect whether predicted risks are numerically accurate. This distinction is clinically important. Clinicians and patients require not only relative risk stratification but also reliable estimates of absolute risk to guide surveillance and treatment decisions. Future studies should therefore report calibration plots, calibration intercepts, calibration slopes, and other relevant calibration measures.

Fifth, clinical utility was inconsistently evaluated. Although some studies reported decision curve analysis, many did not assess whether model-guided decisions would provide net benefit compared with standard clinical strategies. Ultimately, a prediction model is useful only if it improves clinical decision-making. Prospective impact studies are needed to determine whether these models can improve outcomes, reduce unnecessary surveillance, guide adjuvant therapy, or optimize resource allocation.

### 4.6 Strengths and limitations of this review

This review has several strengths. It was conducted in accordance with PRISMA guidance and was prospectively registered. Data extraction was guided by the CHARMS checklist, and risk of bias and applicability were assessed using PROBAST, which is specifically designed for prediction model studies. This framework enabled a structured evaluation of both model characteristics and methodological quality.

The review also adopted a cautious synthesis strategy. Narrative synthesis was prioritized due to heterogeneity in populations, outcomes, prediction horizons, modelling methods, and validation strategies. Meta-analysis was performed only as an exploratory supplement when sufficient validation AUC data were available. This approach reduces the risk of overstating the certainty or generalizability of the findings.

Several limitations should also be acknowledged. First, despite the absence of geographical restrictions, all eligible studies were conducted in East Asian populations. This limits the generalizability of the findings to other regions and healthcare settings. Second, all included studies were retrospective, which introduces risks of selection bias, incomplete follow-up, and unmeasured confounding. Third, recurrence-related outcomes were heterogeneous across studies, limiting direct comparison and quantitative synthesis. Fourth, the number of models eligible for exploratory meta-analysis was limited, and most pooled AUC estimates were derived from internal validation rather than external validation. Fifth, reporting quality was variable, particularly regarding missing data handling, sample size justification, calibration statistics, and complete model specification.

### 4.7 Implications for future research and clinical practice

Future research should place greater emphasis on validation, transparency, and clinical usefulness rather than repeated development of new models using small retrospective datasets. Large multicenter cohorts from diverse geographic regions are needed to evaluate whether existing models maintain performance across different populations, surgical strategies, pathology practices, imaging protocols, and follow-up systems.

Standardized outcome definitions are also essential. Future studies should clearly distinguish postoperative structural recurrence from persistent disease, biochemical incomplete response, DFS,RFS, and site-specific nodal recurrence. The event definition, confirmation method, time origin, and prediction horizon should be explicitly reported.

Model development should follow established reporting and methodological standards, including TRIPOD and PROBAST recommendations. Studies should provide transparent predictor selection procedures, sample size justification, missing data handling strategies, calibration assessment, complete model equations, and clinical utility evaluation. For machine learning and radiomics models, additional attention should be given to reproducibility, feature stability, interpretability, calibration, and external validation.

From a clinical perspective, current prediction models should not replace established clinicopathological risk stratification systems. Their more realistic near-term role is as potential complements to existing frameworks. If adequately validated, they may help refine individualized recurrence risk estimates, particularly in patients with intermediate or uncertain risk classification. For patients, this may enable more tailored follow-up rather than a one-size-fits-all approach. For clinicians, it may support more informed discussions regarding surveillance intensity, additional imaging, or adjuvant therapy. However, such applications require stronger evidence than is currently available.

## 5 Conclusion

Existing prediction models for postoperative recurrence in PTC show potential, but the current evidence base remains insufficient for routine clinical implementation. The field has progressed beyond the identification of isolated risk factors, but it has not yet produced prediction tools that are consistently validated, well calibrated, transparently reported, and demonstrably useful in clinical practice. Future work should therefore prioritize standardized recurrence definitions, rigorous external validation, complete reporting, and prospective impact assessment. Only then can prediction models meaningfully support individualized long-term care for patients with PTC.

## Data Availability

The original contributions presented in the study are included in the article/**Supplementary Material**. Further inquiries can be directed to the corresponding author/s.

## Glossary

ATA: American Thyroid Association
AUS/FLUS: atypia of undetermined significance/follicular lesion of undetermined significance
BMI: body mass index
BRAF: B-Raf proto-oncogene
BRAFneg: BRAF mutation-negative
C-index: concordance index
CHARMS: CHecklist for critical Appraisal and Meta-analysis of prediction Modelling Studies
CI: confidence interval
cN1: clinical N1 disease
DCA: decision curve analysis
DFS: disease-free survival
DTC: differentiated thyroid carcinoma
ENE: extranodal extension
ETE: extrathyroidal extension
FreeT4: free thyroxine
FTC: follicular thyroid carcinoma
IQR: interquartile range
LASSO: least absolute shrinkage and selection operator
LDL: low-density lipoprotein
LN: lymph node
LNM: lymph node metastasis
LNR: lymph node metastasis ratio
MeSH: Medical Subject Headings
N1a: N1a-stage papillary thyroid carcinoma
NMLN: number of metastatic lymph nodes
PICOS: Population, Intervention, Comparator, Outcome, and Setting
PRISMA: Preferred Reporting Items for Systematic Reviews and Meta-Analyses
PROBAST: Prediction model Risk Of Bias ASsessment Tool
PROSPERO: International Prospective Register of Systematic Reviews
PTC: papillary thyroid carcinoma
Rad-scores: radiomics scores
RF: random forest
RFS: recurrence-free survival
ROM: risk of malignancy
STR: structural recurrence
SV: support vector
TBSRTC: The Bethesda System for Reporting Thyroid Cytopathology
Tg: thyroglobulin
TgAb: thyroglobulin antibody
TRIPOD: Transparent Reporting of a multivariable prediction model for Individual Prognosis Or Diagnosis.
