# Supplementary material for: Prediction models for postoperative recurrence in papillary thyroid carcinoma: a systematic review and critical appraisal
Source: Front Endocrinol (Lausanne). 2026 Jul 15;17:1831081. doi: 10.3389/fendo.2026.1831081 (PMC13414811; doi:10.3389/fendo.2026.1831081)
Supplement: Supplementary file 1 [file DataSheet1.pdf]

Literature search strategy.

1.Pubmed

Search number	Query
#1	"thyroid neoplasms"[Title/Abstract] OR "thyroid neoplasms"[MeSH Terms] OR "neoplasm thyroid"[Title/Abstract] OR "thyroid neoplasm"[Title/Abstract] OR "neoplasms thyroid"[Title/Abstract] OR "thyroid carcinoma"[Title/Abstract] OR "carcinoma thyroid"[Title/Abstract] OR "carcinomas thyroid"[Title/Abstract] OR "thyroid carcinomas"[Title/Abstract] OR "thyroid cancers"[Title/Abstract] OR "thyroid cancer"[Title/Abstract] OR "cancer thyroid"[Title/Abstract] OR "cancers thyroid"[Title/Abstract] OR "cancer of the thyroid"[Title/Abstract] OR "thyroid adenoma"[Title/Abstract] OR "adenoma thyroid"[Title/Abstract] OR "adenomas thyroid"[Title/Abstract] OR "thyroid adenomas"[Title/Abstract]
#2	("Recurrence"[MeSH Terms] OR "Recurrences"[Title/Abstract] OR "Recrudescence"[Title/Abstract] OR "Recrudescences"[Title/Abstract] OR "Relapse"[Title/Abstract] OR "Relapses"[Title/Abstract] OR "Recurrence"[Title/Abstract] OR ("local neoplasm recurrences"[Title/Abstract] OR "locoregional neoplasm recurrence"[Title/Abstract] OR "recurrences local neoplasm"[Title/Abstract] OR (("neoplasm s"[All Fields] OR "neoplasms"[MeSH Terms] OR "neoplasms"[All Fields] OR "Neoplasm"[All Fields]) AND "recurrences local"[Title/Abstract]) OR (("recurrance"[All Fields] OR "Recurrence"[MeSH Terms] OR "Recurrence"[All Fields] OR "Recurrences"[All Fields] OR "recurrencies"[All Fields] OR "recurrency"[All Fields] OR "recurrent"[All Fields] OR "recurrently"[All Fields] OR "recurrents"[All Fields]) AND "local neoplasm"[Title/Abstract]) OR (("recurrance"[All Fields] OR "Recurrence"[MeSH Terms] OR "Recurrence"[All Fields] OR "Recurrences"[All Fields] OR "recurrencies"[All Fields] OR "recurrency"[All Fields] OR "recurrent"[All Fields] OR "recurrently"[All Fields] OR "recurrents"[All Fields]) AND "locoregional neoplasm"[Title/Abstract]) OR "local neoplasm recurrence"[Title/Abstract] OR "neoplasm recurrence locoregional"[Title/Abstract] OR "locoregional neoplasm recurrences"[Title/Abstract] OR (("neoplasm s"[All Fields] OR "neoplasms"[MeSH Terms] OR "neoplasms"[All Fields] OR "Neoplasm"[All Fields]) AND "recurrences locoregional"[Title/Abstract]) OR (("neoplasm s"[All Fields] OR "neoplasms"[MeSH Terms] OR "neoplasms"[All Fields] OR "Neoplasm"[All Fields]) AND "recurrences locoregional"[Title/Abstract]) OR ("neoplasm recurrence, local"[MeSH Terms] OR ("Neoplasm"[All Fields] AND "Recurrence"[All Fields] AND "Local"[All Fields]) OR "local neoplasm recurrence"[All Fields] OR "neoplasm recurrence local"[All Fields]) OR "neoplasm recurrence, local"[MeSH Terms])) OR (Recurrence[Title/Abstract])
#3	(prediction model) OR (predictive model)
#4	#1 AND #2 AND #3

2.Cochrane

Search number	Query
#1	MeSH descriptor: [Thyroid Neoplasms] explode all trees
#2	(thyroid neoplasms):ti,ab,kw OR (Neoplasm, Thyroid):ti,ab,kw OR (Thyroid Neoplasm):ti,ab,kw OR (Neoplasms, Thyroid):ti,ab,kw OR (Thyroid Carcinoma):ti,ab,kw OR (Carcinoma, Thyroid):ti,ab,kw OR (Carcinomas, Thyroid):ti,ab,kw OR (Thyroid Carcinomas):ti,ab,kw OR (Cancer of Thyroid):ti,ab,kw OR (Thyroid Cancers):ti,ab,kw OR (Thyroid Cancer):ti,ab,kw OR (Cancer, Thyroid):ti,ab,kw OR (Cancers,

	Thyroid):ti,ab,kw OR (Cancer of the Thyroid):ti,ab,kw OR (Thyroid Adenoma):ti,ab,kw OR (Adenoma, Thyroid):ti,ab,kw OR (Adenomas, Thyroid):ti,ab,kw OR (Thyroid Adenomas):ti,ab,kw
#3	#1 OR #2
#4	MeSH descriptor: [Recurrence] explode all trees
#5	MeSH descriptor: [Neoplasm Recurrence, Local] explode all trees
#6	#4 OR #5
#7	(Recurrence) OR (Recurrences) OR (Recrudescence) OR (Recrudescences) OR (Relapse) OR (Relapses)
#8	(Neoplasm Recurrence, Local) OR (Local Neoplasm Recurrences) OR (Locoregional Neoplasm Recurrence) OR (Recurrences, Local Neoplasm) OR (Neoplasm Recurrences, Local) OR (Recurrence, Local Neoplasm) OR (Recurrence, Locoregional Neoplasm) OR (Local Neoplasm Recurrence) OR (Neoplasm Recurrence, Locoregional) OR (Locoregional Neoplasm Recurrences) OR (Neoplasm Recurrences, Locoregional) OR (Recurrences, Locoregional Neoplasm)
#9	#6 OR #7 OR #8
#10	(prediction model) OR (predictive model)
#11	#3 AND #9 AND #10

3.Embase

Search number	Query
#1	'thyroid tumor'/exp OR 'thyroid tumor'
#2	'thyroid neoplasms':ti,ab,kw OR 'Neoplasm, Thyroid':ti,ab,kw OR 'Thyroid Neoplasm':ti,ab,kw OR 'Neoplasms, Thyroid':ti,ab,kw OR ' Thyroid Carcinoma':ti,ab,kw OR 'Carcinoma, Thyroid':ti,ab,kw OR 'Carcinomas, Thyroid':ti,ab,kw OR 'Thyroid Carcinomas':ti,ab,kw OR 'Cancer of Thyroid':ti,ab,kw OR 'Thyroid Cancers':ti,ab,kw OR 'Thyroid Cancer':ti,ab,kw OR 'Cancer, Thyroid':ti,ab,kw OR 'Cancers, Thyroid':ti,ab,kw OR 'Cancer of the Thyroid':ti,ab,kw OR 'Thyroid Adenoma':ti,ab,kw OR 'Adenoma, Thyroid':ti,ab,kw OR 'Adenomas, Thyroid':ti,ab,kw OR 'Thyroid Adenomas':ti,ab,kw
#3	#1 OR #2
#4	'Recurrence'/exp OR 'Neoplasm Recurrence, Local'/exp
#5	'Recurrence':ti,ab,kw OR 'Recurrences':ti,ab,kw OR 'Recrudescence':ti,ab,kw OR 'Recrudescences':ti,ab,kw OR 'Relapse':ti,ab,kw OR 'Relapses':ti,ab,kw
#6	'Neoplasm Recurrence, Local':ti,ab,kw OR 'Local Neoplasm Recurrences':ti,ab,kw OR 'Locoregional Neoplasm Recurrence':ti,ab,kw OR 'Recurrences, Local Neoplasm':ti,ab,kw OR 'Neoplasm Recurrences, Local':ti,ab,kw OR 'Recurrence, Local Neoplasm':ti,ab,kw OR 'Recurrence, Locoregional Neoplasm':ti,ab,kw OR 'Local Neoplasm Recurrence':ti,ab,kw OR 'Neoplasm Recurrence, Locoregional':ti,ab,kw OR 'Locoregional Neoplasm Recurrences':ti,ab,kw OR 'Neoplasm Recurrences, Locoregional':ti,ab,kw OR 'Recurrences, Locoregional Neoplasm':ti,ab,kw
#7	#4 OR #5 OR #6
#8	'prediction model' OR 'predictive model'
#9	#3 AND #7 AND #8
