# Supplementary figures and images for: Prediction models for postoperative recurrence in papillary thyroid carcinoma: a systematic review and critical appraisal
Source: Front Endocrinol (Lausanne). 2026 Jul 15;17:1831081. doi: 10.3389/fendo.2026.1831081 (PMC13414811; doi:10.3389/fendo.2026.1831081)

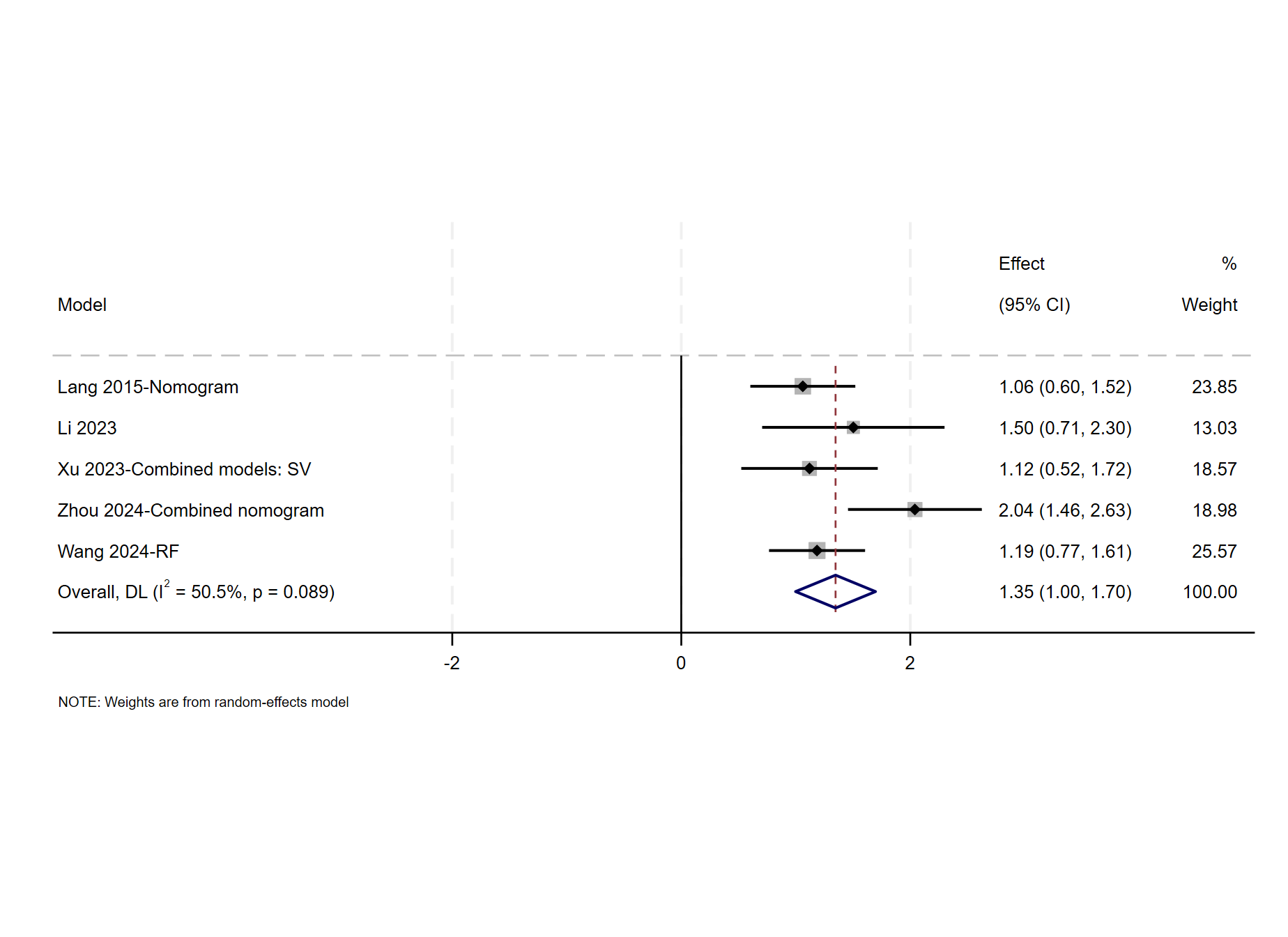

Supplement: Supplementary file 2 [file Image1.tif]
